# TAFRO syndrome with renal biopsy successfully treated with steroids and cyclosporine: a case report

**DOI:** 10.1186/s12882-022-02886-5

**Published:** 2022-07-23

**Authors:** Takahide Iwasaki, Kosuke Mizusaki, Miwa Masumoto, Yuko Minagawa, Kouta Azuma, Tetsuya Furukawa, Makoto Yoshida, Takahiro Kuragano

**Affiliations:** 1 Division of Kidney and Dialysis, Hyogo College of Medicine, 1-1 Mukogawa-cho, Nishinomiya City, Hyogo 663-8501 Japan; 2 Division of Allergology and Rheumatology, Hyogo College of Medicine, Mukogawa-cho, Nishinomiya City, Hyogo 663-8501 Japan; 3 Division of Surgical Pathology, Hyogo College of Medicine, Mukogawa-cho, Nishinomiya City, Hyogo 663-8501 Japan

**Keywords:** TAFRO syndrome, Kidney biopsy, Thrombotic microangiopathy, Cyclosporin, Sjogren’s syndrome

## Abstract

**Background:**

TAFRO syndrome is an acute or subacute systemic inflammatory disease with no apparent cause, presenting with fever, generalized edema, thrombocytopenia, renal damage, anemia, and organ enlargement. Interleukin-6, vascular endothelial growth factor, and other cytokines are thought to be the etiologic agents that increase vascular permeability and cause the resulting organ damage. Only few reports of renal biopsy performed in patients with TAFRO syndrome exist.

**Case presentation:**

A 61-year-old woman, with a history of Sjogren’s syndrome, was admitted to our hospital with anasarca and abdominal distension. Based on the clinical course and various laboratory findings, we diagnosed TAFRO syndrome. Renal biopsy revealed thrombotic microangiopathy, including endothelial cell swelling, subendothelial space expansion, and mesangiolysis. She was treated with oral prednisolone and cyclosporine, with consequent resolution of anasarca, pleural effusion, and ascites, and improvement in renal function and urinary findings. The patient’s platelet count also normalized after 2 months of treatment.

**Conclusions:**

Given that only few reports of improvement in the systemic symptoms of TAFRO syndrome using steroids and cyclosporine exist, our study investigating the relationship between the pathogenesis of TAFRO syndrome and renal disorders, as well as treatment methods, provides valuable insights.

## Background

TAFRO syndrome is an acute or subacute systemic inflammatory disease with no apparent cause, manifesting as thrombocytopenia (T), anasarca (A) (pleural effusion and ascites), fever (F), reticulin fibrosis (R) (renal impairment), and organomegaly (O) (organ enlargement: hepatosplenomegaly and lymph node enlargement). In 2012, TAFRO syndrome was proposed in Japan as an independent syndrome comprising thrombocytopenia, anasarca, fever, reticulin fibrosis or renal insufficiency, and organomegaly [1, 2]. Subsequently, diagnostic criteria have been proposed for TAFRO syndrome [3, 4] to include three essential criteria—(1) fluid retention, (2) thrombocytopenia, and (3) unexplained fever or positive inflammatory reaction—and two minor criteria—(1) Castleman’s disease-like findings on lymph node biopsy, (2) bone marrow fibrosis or megakaryocytes, and (3) mild. In TAFRO syndrome, elevated levels of cytokines, including interleukin-6 (IL-6) and vascular endothelial growth factor (VEGF), are thought to induce inflammation and vascular permeability, leading to organ damage. TAFRO syndrome is also thought to be secondary to hypercytokinemia caused by infection, collagen disease, or malignancy. However, there is no established treatment, although appropriate diagnosis and prompt treatment are necessary because of the rapid progression of systemic symptoms. Steroids, cyclosporine, tocilizumab, and rituximab are thought to be effective [3]. As TAFRO syndrome is a rare and relatively new disease concept and thrombocytopenia is often observed, only few reports are available on renal biopsy results. However, the frequency of renal injury in TAFRO syndrome is as high as 50% [3]; therefore, it is necessary to study more cases in the future.

Herein, we report a case of TAFRO syndrome associated with Sjogren’s syndrome in which renal biopsy was performed and prednisolone and cyclosporine therapy was effective.

## Case presentation

A 61-year-old woman being managed for Sjogren’s syndrome at the Department of Internal Medicine for Rheumatology became aware of anasarca and abdominal distension about one month ago. Her weight had also increased by approximately 6 kg. She was admitted to our department because her blood tests showed renal dysfunction and abnormalities on urinalysis. Blood tests that had been performed one month earlier showed no evidence of renal dysfunction (creatinine = 0.78 mg/dl), and urinalysis showed no abnormalities. She had never been diagnosed with renal dysfunction and had not shown abnormalities on urine analysis at a previous hospital.

On admission, her clinical findings were as follows: blood pressure, 114/74 mmHg; pulse rate, 94/min; body temperature, 38.2 °C; height, 156 cm; and weight, 46 kg. She was alert with mild facial, upper and lower extremity, and abdominal distension due to edema. She had no superficial lymphadenopathy, joint pain, neurological findings, or skin lesions, and an examination of her heart and lungs showed unremarkable findings.

Laboratory findings on admission are shown in Table 1. Renal function was mildly impaired (creatinine = 1.07 mg/dL, eGFR = 41 mL/min/1.73 m^2^). Urinalysis showed urine protein 2 + [urinary protein-to-creatinine ratio (UTP/Ucr): 0.78 g/gCr] and microscopic hematuria. Antinuclear antibodies (speckled pattern) × 40 and anti-SS-A antibodies showed positive results. Biochemical tests showed low platelet count (7.2 × 10^4^/μL) and high C-reactive protein (CRP) (3.33 mg/dL) level. Tests for hepatitis B, hepatitis C, human immunodeficiency virus, human herpesvirus 8 (HHV-8), and tuberculosis infection were negative. Both serum IL-6 level at 14.8 pg/mL (reference value: 0.0–4.0 pg/mL) and serum VEGF at 384 pg/mL (reference value: 0.0–38.3 pg/mL) were elevated. Haptoglobin showed no decrease, and blood smears showed no crushed red blood cells. ADAMTS13 (a disintegrin-like and metalloproteinase with thrombospondin type 1 motifs 13) activity did not decrease to 34%, and the diagnosis of thrombotic thrombocytopenic purpura (TTP) was negative.

**Table1:** Laboratory findings on admission

Parameter	Value (reference range)	Parameter	Value (reference range)
Hematology		Coagulation	
WBC	12,050/μL (4000–9000)	INR	1.16 (0.85–1.15)
RBC	291 × 10^4^/μL (380–500 × 10^4^)	APTT	46.1 s (26.1–46.1)
Hb	8.7 g/dL (11.5–15.0)	Fib	420 mg/dl (150–450)
Ht	27.3% (35.0–46.0)	D-dimer	4.59 μg/dl (< 0.5)
PLT	7.2 × 10^4^/μL (15.0–35.0 × 10^4^)		
		Serology	
Biochemistry		IgG	2278 mg/dl (870–1700)
TP	7.0 g/dL (6.6–8.1)	IgA	132 mg/dl (110–410)
Alb	2.8 g/dL (4.1–5.1)	IgM	158 mg/dl (35–220)
AST	20 IU/L (13–30)	C3	95 mg/dl (86–160)
ALT	8 IU/L (7–23)	C4	20 mg/dl (17–45)
ALP	92 IU/L (38–113)	CH50	54.8U/ml (31.6–57.6)
GTP	15 IU/L (9–32)	ADAMTS13	34% (> 10)
LDH	194 IU/L (124–222)	IL-6	14.8 pg/ml (0–4.0)
BUN	29 mg/dl (8–20)	VEGF	384 pg/ml (0–38.3)
Cr	1.07 mg/dl (0.46–0.79)	ANA	40 (< 40)
eGFR	41 ml/min/1.73m^2^	SS-A Ab	821U/ml (0–10.0)
UA	8.0 mg/dl (2.6–7.0)	SS-B Ab	4.9U/ml (0–10.0)
Na	136 mEq/L (138–145)	MPO-ANCA	< 1.0EU (< 1.0)
K	4.4 mEq/L (3.6–4.8)	PR3-ANCA	< 1.0EU (< 1.0)
Cl	101 mEq/L (101–108)	s-IL2R	2046U/ml (122–496)
CRP	3.33 mg/dl (< 0.3)	Haptoglobin	212 mg/dl (15–116)
Glucose	98 mg/dl (70–109)	Cryoglobulin	-
HbA1c	4.6% (4.6–6.2)	TSPOT	-
		HHV8	-
Urinalysis		syphilis	-
Protein	2 +	HBs Ag	-
Occult blood	-	HCV Ab	-
UTP/UCr	0.78 g/gCr	HIV	-
β2MG	316 ng/ml (< 150)	M protein	negative
BJP	negative		

**Fig. 1:**
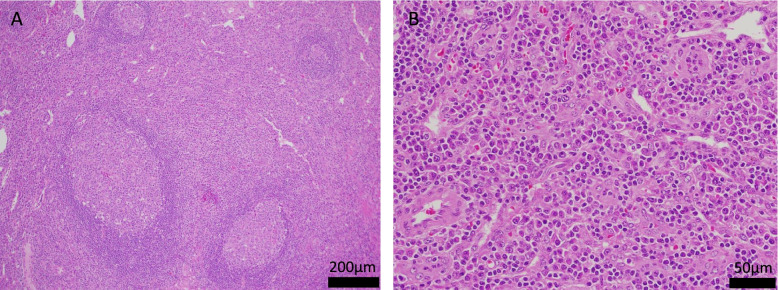
**a** Lymphatic tissue with preserved follicular structure and enlarged interfollicular spaces (hematoxylin and eosin staining, × 100). **b** Prominent plasmacytoid cells between follicles in lymph nodes. The vitrified blood vessels between the follicles are inconspicuous (hematoxylin and eosin staining, × 400)

**Fig. 2:**
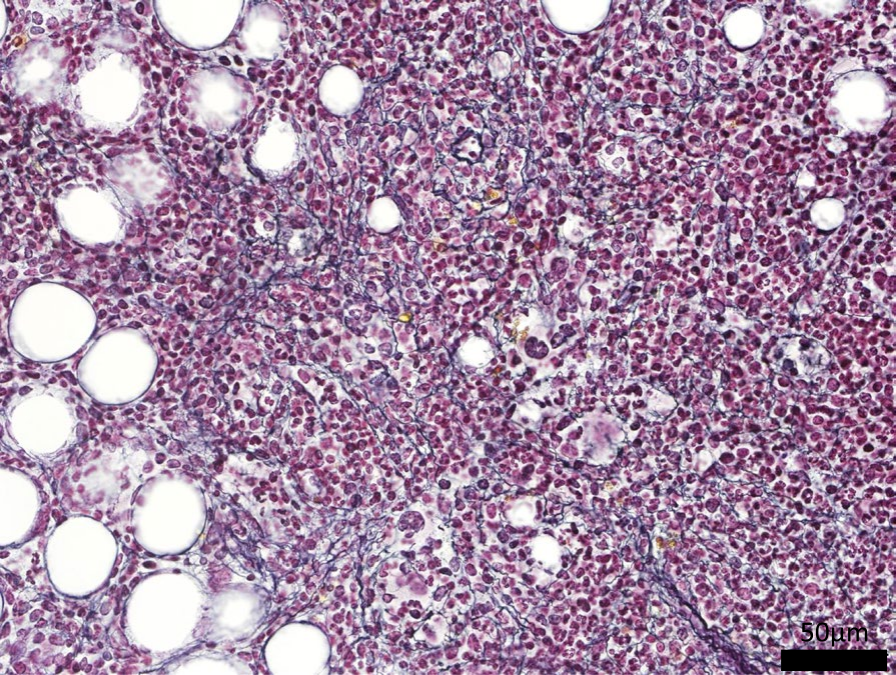
Bone marrow showing extensive mild fibrosis (silver staining, × 400)

Computed tomography revealed generalized lymphadenopathy, pleural effusion, and ascites. A lymph node biopsy (left cervical lymph node) was performed due to the presence of systemic lymphadenopathy. Lymphatic pathology showed lymph nodes with preserved follicular structure and plasmacytoid infiltration between the follicles (Fig. 1a-b). This finding was consistent with lymph node findings in Castleman’s disease. A bone marrow biopsy was also performed due to low platelet count, and extensive fibrosis of the bone marrow was reported (Fig. 2; silver stain was performed to assess fibrosis of the bone marrow). Based on these findings, the patient was diagnosed with TAFRO syndrome (diagnostic criteria: fulfillment of 3 essential and 4 sub-criteria).

**Fig. 3:**
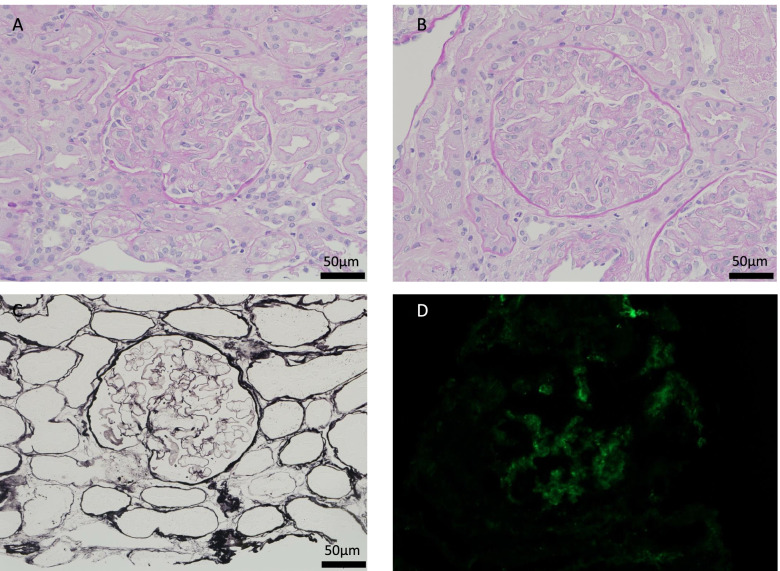
**a**-**c** Light microscopy showing diffuse global endocapillary proliferative changes with endothelial swelling and expansion of the subendothelial space in the glomerulus (periodic acid–Schiff staining, × 400). **c** Light microscopy showing no evidence of double contours in capillary walls or spike (periodic acid-methenamine silver staining, × 400). **d** Immunofluorescence studies revealing mesangial deposits of only IgM in the glomerulus

**Fig. 4:**
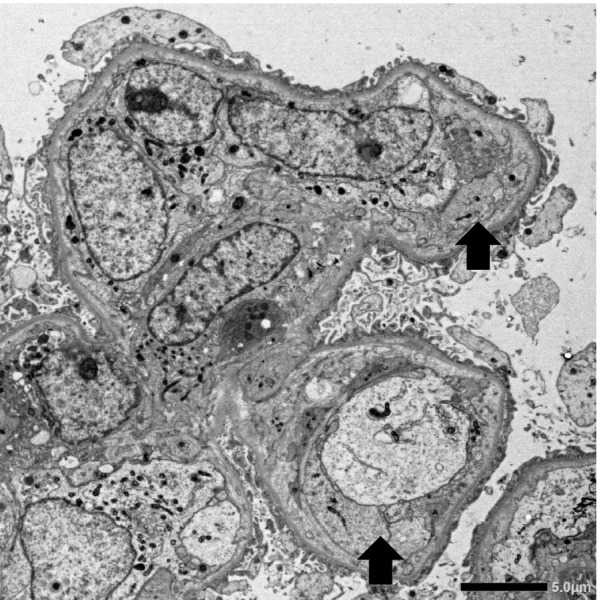
Electron microscopy showing endothelial cell swelling with expansion of the subendothelial space (arrows)

A renal biopsy was also performed to further assess renal dysfunction and abnormal urine analysis results. Interstitial nephritis associated with Sjogren’s syndrome was suggested as one of the differential causes of renal damage. Periodic acid–Schiff staining revealed diffuse endothelial cell swelling and macrophages in the glomerulus (Fig. 3a-b). Periodic acid-methenamine staining showed no double contours or spike (Fig. 3c). No areas of tubular interstitium were found to be devitalized, and no inflammatory cell infiltration was observed. There was no fibrin thrombus or fibrinoid necrosis in the glomeruli or arterioles. Immunofluorescence staining showed that only IgM was deposited in the mesangial region, whereas other immunoglobulins and complement were not (Fig. 3d). Congo red stain was performed to search for renal amyloid, but the results were negative. Electron microscopy revealed endothelial cell swelling, subendothelial space enlargement, and mesangiolysis, but showed no electron-dense deposits (Fig. 4). Therefore, interstitial nephritis due to Sjogren’s syndrome was ruled out, and thrombotic microangiopathy (TMA) due to TAFRO syndrome was diagnosed.

Optical microscopy (lymph node biopsy, bone marrow biopsy, and renal biopsy) was performed using an Olympus BX53(Olympus, Tokyo, Japan). The imaging software used was cellsens standard(Olympus, Tokyo, Japan). Image resolution is 4080 × 3072. Immunofluorescence staining and electron microscopy of kidneys were performed by SRL(Tokyo, Japan), a private laboratory.

**Fig. 5:**
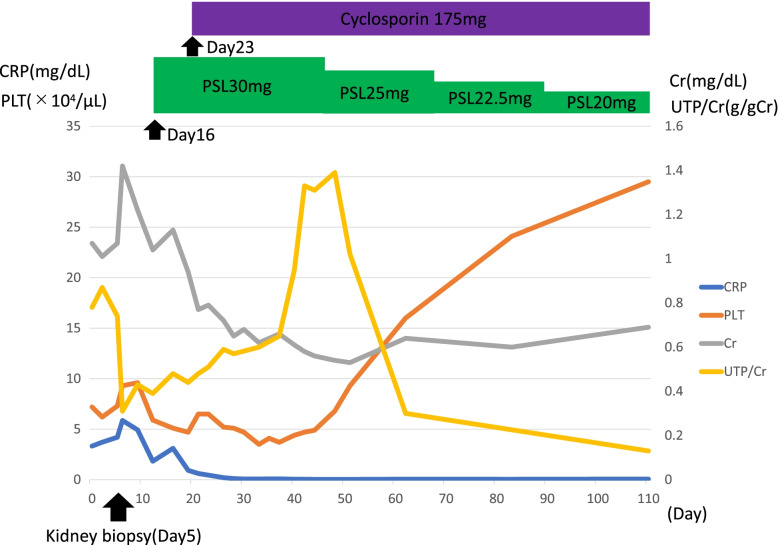
The patient’s clinical course. Abbreviations: CRP: C-reactive protein; PLT: Platelet; Cr: Creatinine; UTP: Urinary Total protein; PSL: Prednisolone

The diagnosis of TAFRO syndrome was made on the 16th day after admission, and treatment with prednisolone 30 mg was started. Since the patient had markedly low platelet count with massive pleural effusion and ascites, additional treatment with 175 mg of cyclosporine was started on the 23rd day after admission to intensify the treatment. The clinical course is shown in Fig. 5. Fever and CRP level improved immediately after initiation of treatment. Renal dysfunction and urinary protein also normalized relatively early. The platelet count increased gradually from one month after initiation of treatment and normalized after approximately two months. Treatment for anasarca, pleural effusion, and ascites was initiated using 4 mg of torasemide (a diuretic); however, no improvement was observed. Therefore, steroids and cyclosporine were commenced, and resolution was noted after approximately two months.

## Discussion and conclusions

We believe that this report is important because TAFRO syndrome is a rare disease and renal biopsy is often avoided in many institutions due to severe thrombocytopenia. In addition, to the best of our knowledge, there are no case reports of the use of cyclosporine in patients who have undergone renal biopsies due to renal impairment.

There are a few reports on the renal pathological features of TAFRO syndrome. In previous case reports, the renal pathology of TAFRO syndrome included TMA [5–8] or membranous proliferative glomerulonephritis (MPGN) [9, 10]. Patients with relatively early renal biopsy tended to have TMA, while those with late renal biopsy tended to have MPGN, suggesting that MPGN lesions in TAFRO syndrome result from chronic TMA lesions. In addition, TMA in TAFRO syndrome is characterized by the absence of fibrinoid necrosis and fibrin thrombus, unlike the usual TMA [TTP, hemolytic uremic syndrome (HUS), atypical HUS, and secondary TMA].

In 2020, Mizuno et al. reported a case series of seven cases of TAFRO syndrome in which renal biopsy was performed [11]. Clinically, the patients presented with severe diuretic-resistant anasarca, mild proteinuria, and renal dysfunction. Blood tests showed a median IL-6 of 12.3 pg/ml and a median VEGF of 177 pg/ml, which were high as in the present case. Renal biopsy showed endothelial cell swelling, mesangiolysis or loosening of the mesangium, and double contour and thickening of the glomerular basement membrane. Electron microscopy showed loss of mesangial structures with subendothelial space enlargement, endothelial cell swelling, and loss of fenestra. The renal pathological findings reported by Mizuno et al. were similar to those reported in previous case reports [5–8]; however, in our study, arteriolar myocyte vacuolization was newly reported as a characteristic renal pathological finding in TAFRO syndrome.

The fluorescent staining findings of kidney tissues in TAFRO syndrome are reported to be negative for all immunoglobulins and complement, thus helping to rule out immune complex nephritis [5–10]. A rare case of IgM positivity has been reported, but it was thought to be associated with mesangial matrix and subendothelial space exudative lesions induced by endothelial cell damage [12]. In our case, IgM was also deposited on the mesangium, as noted on immunofluorescence staining. Based on the light microscopy and immunofluorescent staining findings, we believe that there was strong endothelial cell damage in the glomerulus in our case.

Overproduction of VEGF induced by IL-6 can lead to glomerular endothelial injury [13]. VEGF plays a key role in the regulation of the structure and function of endothelial cells [14], and in TAFRO syndrome blood levels of VEGF are elevated thereby inducing endothelial cell damage in the glomerulus.

VEGF is also overexpressed in diabetics [15]. Excess VEGF downregulates nephrin, the filtration membrane of the glomeruli in the kidney, causing glomerular disease and proteinuria [16]. Examination of diabetic mouse models suggests that the severity of diabetic nephropathy is determined by renal local VEGF levels rather than by systemic VEGF levels [17]. D Veron et al. examined the pathogenicity of VEGF overexpression in podocytes [18]. In mice, induction of podocyte-specific VEGF overexpression elicited glomerular damage. The study by Veron et al. also established that VEGF receptor-2 (VEGFR2) is expressed in podocytes and glomerular endothelial cells, confirming the interaction between VEGFR2 and nephrin. This suggests that autocrine or paracrine VEGF signaling mediated by VEGFR2 may occur in podocytes and induce glomerular damage due to VEGF overexpression.

Locally, VEGF is produced in podocytes and has been shown to inhibit glomerular endothelial cell damage in the kidney [19]. It has also been shown that the administration of bevacizumab, a monoclonal antibody to VEGF, induces TMA in the kidney [20]. In short, these studies suggest that focal renal VEGF expression suppresses TMA and plays a renoprotective role. Thus, whether VEGF induces renal injury or plays a protective role is controversial and requires further study.

Currently, the ideal treatment strategy for patients with TAFRO syndrome has not been established. High-dose steroids, cyclosporine, tocilizumab, and rituximab are commonly used, but these therapies often result in treatment failure and relapse. Shimada et al. reported a case series of the treatment of three patients with TAFRO syndrome who underwent renal biopsy [21]. All patients had TMA, as revealed by renal pathology findings; two patients were treated with steroids or cyclosporine, while the steroid-refractory patient was refractory to tocilizumab despite high serum IL-6 levels. Similar cases of patients resistant to tocilizumab therapy have been reported previously [22]. While the use of tocilizumab in TAFRO syndrome with high levels of the pro-inflammatory cytokine IL-6 seems appropriate, humoral factors other than IL-6 may also be involved in the pathogenesis of TAFRO syndrome, as some patients are refractory even to treatment with tocilizumab.

In our case, cyclosporine was added to the steroids because of the marked thrombocytopenia in addition to renal impairment. The platelet count normalized with the addition of cyclosporine. Allegra et al. reported the efficacy of cyclosporine for thrombocytopenia in TAFRO syndrome [23]. Cyclosporine, which inhibits T-cell function by inhibiting IL-2, is one of the treatment options for patients with TAFRO syndrome who are resistant to tocilizumab [24]. IL-2 as well as IL-6 may contribute to the inflammatory cytokines involved in TAFRO syndrome. When IL-2 is used in cancer treatment, it reportedly may induce symptoms similar to TAFRO syndrome, such as microangiopathy, renal dysfunction, and thrombocytopenia [25]. However, there are few reports on the treatment of TAFRO syndrome with steroids and cyclosporine, and therefore further accumulation of data is needed.

Only few reports of combined cases of Sjogren’s syndrome and TAFRO syndrome exist in the literature [26]. TAFRO syndrome may occur secondary to an infection, collagen disease, or malignancy. The patient in our study might have developed TAFRO syndrome secondary to Sjogren’s syndrome, or it might simply have been an incidental complication. In cases of Sjogren’s syndrome combined with TAFRO syndrome, renal involvement must be differentiated from interstitial nephritis. In our study, no inflammatory cell infiltration into the interstitial space was observed on renal pathology. Therefore, if patients present with interstitial lesions, the diagnosis of renal pathology might be more difficult.

In summary, despite being a rare disease, remission in TAFRO syndrome can be achieved with appropriate treatment. Additionally, the patient in our study was able to complete tests that required blood observation procedures (renal biopsy, lymph node biopsy, and bone marrow biopsy) before thrombocytopenia, allowing for an early diagnosis. The diagnosis of TAFRO syndrome before the onset of renal failure due to TMA allows the use of cyclosporine when marked thrombocytopenia develops. It is therefore important to diagnose TAFRO syndrome early and initiate appropriate treatment before the disease becomes severe.

## Data Availability

The datasets used and/or analyzed during the current study are available from the corresponding author on reasonable request.

## Abbreviations

ADAMTS13: A disintegrin-like and metalloproteinase with thrombospondin type 1 motifs 13
Cr: Creatinine
CRP: C-reactive protein
HHV-8: Human herpesvirus 8
(HUS): Hemolytic uremic syndrome
Ig: Immunoglobulin
IL-6: Interleukin-6
MPGN: Membranous proliferative glomerulonephritis
PLT: Platelet
PSL: Prednisolone
TMA: Thrombotic microangiopathy
TTP: Thrombotic thrombocytopenic purpura
UTP: Urinary Total protein
VEGF: Vascular endothelial growth factor
